# Short-term eplerenone for treatment of chronic central serous chorioretinopathy; a prospective study

**DOI:** 10.1186/s40942-019-0190-y

**Published:** 2019-09-09

**Authors:** Hamid-Reza Moein, Lauren W. Bierman, Eduardo A. Novais, Carlos Moreira-Neto, Caroline R. Baumal, Adam Rogers, Jay S. Duker, André J. Witkin

**Affiliations:** 1 New England Eye Center, Tufts Medical Center, 800 Washington Street, Box 450, Boston, MA 02111 USA; 2 School of Medicine, Federal University of São Paulo, São Paulo, Brazil; 3 Hospital de Olhos do Parana, Curitiba, Brazil

**Keywords:** Central serous chorioretinopathy, Eplerenone, Mineralocorticoid inhibitors

## Abstract

**Background:**

Increased mineralocorticoid activity is one of the plausible causes of chronic central serous chorioretinopathy (CSCR) and mineralocorticoid inhibitors such as eplerenone have been investigated as its potential therapy. This study investigates the short-term safety and efficacy of oral eplerenone in patients with chronic CSCR.

**Patients and methods:**

Prospective study of 13 eyes of 13 patients with the diagnosis of chronic CSCR. All patients received eplerenone 50 mg/day for 4 weeks. Enhanced depth imaging optical coherence tomography (OCT) was obtained. Best corrected visual acuity (BCVA), and OCT parameters including sub retinal fluid (SRF), choroidal thickness (CT) and central macular thickness (CMT), were measured manually.

**Results:**

The mean SRF height decreased slightly at 1-month follow-up as compared to baseline, but the change was not statistically significant (94.18 ± 17.53 vs. 113.15 ± 18.69; p = 0.08). Subfoveal CT and CMT was significantly reduced as compared to baseline (6.6% [p = 0.002] and 7.05% [p = 0.04], respectively). The BCVA did not change significantly (20/28 vs. 20/30 [p = 0.16]).

**Conclusion:**

This study suggests that oral eplerenone may be used as a safe and potentially effective treatment in chronic CSCR, however there are minimal short-term effects on subretinal fluid or visual acuity therefore therapeutic trials longer than one month are necessary to test its benefits.

*Trial registration* Clinicaltrials.gov identification number: NCT01822561. Registered 3/25/13, https://clinicaltrials.gov/ct2/show/study/NCT01822561

## Introduction

Central serous chorioretinopathy (CSCR) is a disease of the choroid and retina that causes subretinal fluid (SRF) accumulate underneath the macula [1]. CSCR affects 1 in 10,000 individuals [2], more commonly in Asian and Caucasian populations [3]. CSCR is thought to be caused by an underlying abnormality in choroidal vascular permeability along with a dysfunctional or overburdened retinal pigmented epithelium (RPE) pump. This leads to an acute, often self-limiting build-up of serous fluid under the retina, which represents the typical and characteristic feature of CSCR [3, 4].

Optical coherence tomography (OCT) is used for diagnosing CSCR, monitoring disease activity, and quantifying retinal thickness and amount of SRF over time [5]. In the acute phase, SRF often gets spontaneously resorbed over 6–12 weeks [3] or 12–24 weeks [6]. The definition of chronic CSCR is debatable, but it is most commonly defined as SRF fluid lasting more than 3 months, or recurrence of the disease within 1 year [3, 4]. Despite favorable visual acuities, CSCR patients are rarely asymptomatic [7, 8]. In addition, approximately 30–50% of all CSCR patients (treated or untreated) have recurrences of the disease, either in the same eye, opposite eye, or both eyes [3, 4, 8].

Increased mineralocorticoid activity has been suggested as one of the plausible causes of CSCR [9, 10]. Therefore, mineralocorticoid inhibitors have been investigated as a potential therapy for CSCR [9, 11]. Eplerenone is of particular interest, as this medication does not have significant anti-testosterone side effects [12]. Recently published double-blind, randomized, placebo-controlled studies showed contradicting results on the effect of eplerenone on CSCR [12–14] and a new one is underway. This paper describes the short-term (4-week) efficacy of eplerenone in patients with chronic CSCR in a prospective interventional study.

## Patients and methods

This study (Clinicaltrials.gov identification number: NCT01822561) was approved by the Institutional Review Board (IRB) of Tufts Medical Center, Boston, MA, USA. The study was adherent to the Health Insurance Portability and Accountability Act of 1996 and was compliant with tenets of the Declaration of Helsinki.

Patients ≥ 18-years-old with diagnosis of acute or chronic CSCR were recruited between August 2013 to August 2015 from New England Eye Center at Tufts Medical Center. CSCR was diagnosed based on characteristic clinical and imaging findings. Chronic CSCR was defined as patients with previous diagnosis of CSCR and persistent SRF on OCT for ≥ 3 months after the onset of symptoms. Only one eye from each patient was studied and if the patient had CSCR in both eyes only the worse eye (based on the OCT imaging) was included.

The exclusion criteria were: pregnant women or those who were actively trying to conceive, patients with concomitant eye diseases that affect the macula (e.g., age-related macular degeneration and diabetic macular edema), type 2 diabetes with microalbuminuria, serum potassium ≥ 5.0 mEq/L, creatinine > 2 mg/dL in men and > 1.8 mg/dL in women, creatinine clearance < 50 mL/min, concomitant administration of potassium supplements, potassium-sparing diuretics, and/or potent CYP3A4 inhibitors (e.g., amifostine, ketoconazole, and rituximab).

The purpose of the study, details of examinations and testing, and explanations of alternative treatment options including focal laser therapy and/or PDT was described for recruited patients and written consent was obtained. Patients’ medical history, previous treatments for CSCR and current medications were acquired. Within 1 week prior to initiation of treatment, patients were required to have blood pressure measurement as well as full laboratory workup.

**Fig. 1:**
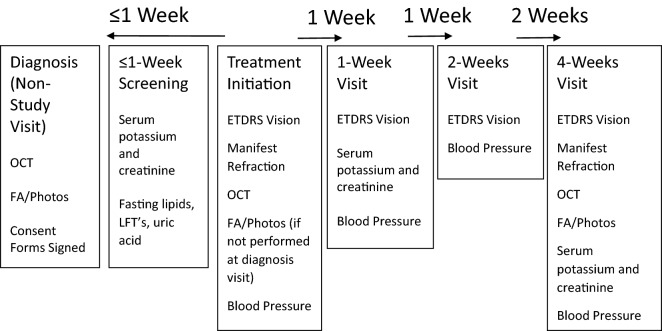
Study protocol. *ETDRS* Early Treatment Diabetic Retinopathy Study, *OCT* optical coherence tomography, *LFT* liver function tests, *FA* fluorescein angiography

If patients qualified for the study and wished to proceed with treatment, they underwent ocular examination (day 1 of study), color fundus photographs, fluorescein angiography, and macular OCT. Patients were given 30 tablets of eplerenone 50 mg on that day and were asked to take one tablet daily for 30 days. Patients were then evaluated at week 1, week 2, and week 4 after treatment initiation. At each visit, patients had a complete ophthalmic exam (including visual acuity, slit lamp, and dilated fundus examination), and blood pressure measurement. Serum creatinine and potassium were re-measured at week 1 and week 4 to detect any potential side effect. Ocular imaging studies and lab work were repeated at week 4. See Fig. 1 for a summary of design protocol.

**Fig. 2:**
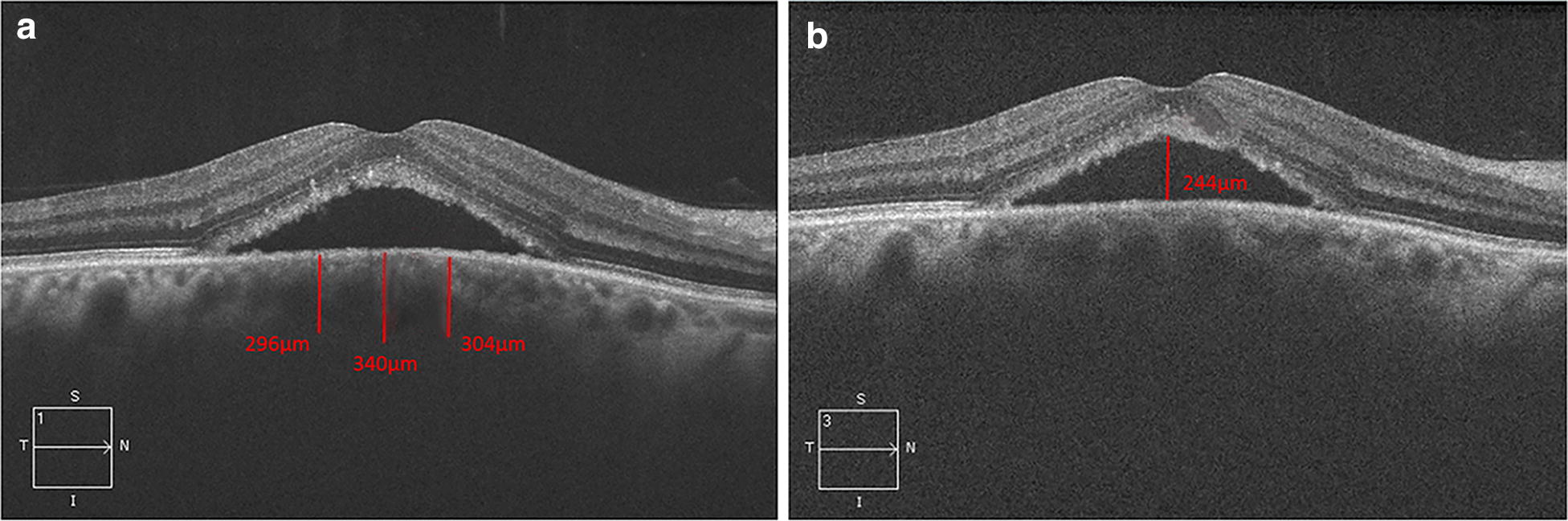
Manual measurement of choroidal thickness and subretinal fluid in a 47 years-old man with acute central serous chorioretinopathy. Measurement tool in Cirrus HD-OCT software (Carl Zeiss Meditec, Dublin, CA) was used for this purpose. **a** A perpendicular line was drawn between outer edge of hyperreflective retinal pigment epithelium (RPE) and the inner sclera. Nasal and temporal choroidal thickness was calculated in a similar fashion at 500 μm intervals nasal and temporal to the fovea, respectively. **b** A perpendicular line was drawn between the neurosensorial retina (inner portion of outer photoreceptor segment) and the RPE, and the maximum height was recorded

Best-corrected visual acuity (BCVA) was measured using the Early Treatment Diabetic Retinopathy Study (ETDRS) chart and converted to logarithm of the minimum angle of resolution (logMAR) for further analysis. OCT images were acquired using Cirrus HD-OCT (Carl Zeiss Meditec, Dublin, CA). Enhanced Depth Imaging (EDI) scans, 5 lines raster scans, and 512 × 128 macular cube scans were obtained, and central macular thickness (CMT) was measured automatically via the OCT software. Baseline and follow up OCT scans were masked, and CT and the maximum height of subretinal fluid (SRF) were manually measured on EDI-OCT scans using the linear measurement tool [15]. A perpendicular line was drawn between the outer edge of the retinal pigment epithelium (RPE) and the choroidal/scleral junction. Nasal and temporal CT were calculated in a similar fashion at 500 μm intervals nasal and temporal to the fovea, respectively (Fig. 2a). SRF under the fovea was measured manually on OCT scans by drawing a perpendicular line between the neurosensorial retina and the inner edge of the RPE, and the maximum measurement (in microns) was reported (Fig. 2b). Any potential side effects of the medication were also recorded at each visit and reported to the IRB.

### Statistical analysis

Data are presented as mean ± standard error of the mean (SEM). D’Agostino and Pearson omnibus normality test was performed to evaluate the distribution pattern of the data. The comparison between the baseline and follow-up measurements were done by Wilcoxon signed rank test and *p* value of < 0.05 was considered as significant. A Spearman correlation test was used to assess the correlation between OCT parameters and demographics with visual acuity. Prism version 6.01 (GraphPad Software, Inc. La Jolla, CA, USA) was used for analysis of the data.

## Results

Fifteen patients were recruited but 13 patients completed the study. Mean duration of symptoms prior to study in these 13 patients was 17.40 ± 3.9 months. Five patients received previous treatments for CSCR. Three patients received intravitreal bevacizumab at 1, 11, and 34 months before eplerenone treatment, respectively. One patient received photodynamic laser therapy (PDT) 9 months prior to starting eplerenone, and another patient had focal laser therapy 4 months prior (Table 1). Thirteen patients completed a 4-week course of the treatment. Two out of thirteen patients continued the treatment by their own request for total of 7 and 20 weeks. The final visit exam results for these 2 patients are reported separately in this section but for the purpose of statistical analysis only the results from week 4 were included (Tables 2, 3).

**Table 1:** Demographics of patients with central serous chorioretinopathy and their previous treatments

Age (years)	55.61 ± 2.32 (45–71 years)
Sex (male/female)	13/0
Duration of CSCR symptoms prior to eplerenone therapy (months)	17.40 ± 3.9 (4–36 months)
Total number of patients with prior treatments	5/13^a^
Photodynamic laser therapy (PDT)	3/13
Focal laser therapy	2/13
Intravitreal bevacizumab	3/13

^a^One patient had received all 3 modes of treatments and one had received 2 modes of treatments before starting eplerenone

**Table 2:** Mean changes in OCT parameters and visual acuity

	Baseline	4 weeks after treatment	p value
Subretinal fluid height (μm)	113.15 ± 18.69	94.18 ± 17.53	0.08
Visual acuity, LogMAR (Snellen equivalent)	0.18 ± 0.08 (20/30)	0.15 ± 0.08 (20/28)	0.16
Nasal choroidal thickness (μm)	410.00 ± 20.36	394.89 ± 17.22	0.14
Subfoveal choroidal thickness (μm)	452.07 ± 19.70	422.20 ± 18.23	0.002
Temporal choroidal thickness (μm)	411.07 ± 21.17	395.96 ± 15.69	0.33
Central macular thickness (μm)	365.23 ± 26.83	339.46 ± 27.29	0.04

**Table 3:** Laboratory values and blood pressure records in studied patients

	Baseline	4 weeks after treatment	p value
Serum potassium (mEq/L)	4.27 ± 0.11	4.38 ± 0.07	0.11
Serum creatinine (mg/dL)	0.92 ± 0.04	0.97 ± 0.06	0.82
Systolic blood pressure (mmHg)	134.76 ± 5.44	127.69 ± 4.55	0.10
Diastolic blood pressure (mmHg)	86.61 ± 2.71	83.00 ± 2.91	0.31

**Fig. 3:**
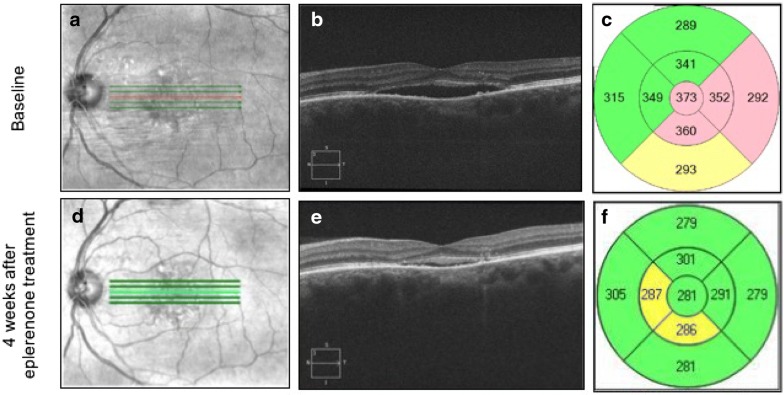
A 58-years-old man with chronic central serous chorioretinopathy in the left eye. He had focal laser therapy 4 months before starting eplerenone therapy. Eplerenone treatment decreased subretinal fluid, choroidal thickness, and central macular thickness after 4 weeks. **a**–**c** Baseline scans before start of eplerenone. **d**, **e** Scans from 4 weeks after eplerenone treatment. **a**, **d** Infrared scans. **b**, **e** 5-line raster enhanced depth-optical coherence tomography (EDI-OCT) from the fovea. **c**, **f** Central macular thickness calculated automatically by the software

Mean SRF height was decreased at 1-month follow-up as compared to baseline, although this did not reach statistical significance (94.18 ± 17.53 μm vs. 113.15 ± 18.69 μm; p = 0.08). However, 4 patients had increased SRF height (ranged 3–30 μm) after 4 weeks of eplerenone treatment. Of note, all of these 4 patients had previous treatments (focal laser therapy, PDT, and/or intravitreal bevacizumab) for CSCR and the patient with maximum increase in SRF height (30 μm) had received all 3 modes of treatments before. Mean subfoveal CT and mean CMT decreased significantly after 4 weeks of treatment as compared to the baseline (Table 2). Mean of laboratory values and blood pressure readings at baseline and after 4 weeks of treatment are also presented in Table 3. EDI-OCT scans and central macular thickness measures in a representative CSCR patient before and after eplerenone treatment is illustrated in Fig. 3.

The patient who continued eplerenone for 20 weeks had complete resolution of SRF whereas the patient who continued intervention for a total of 7 weeks experienced an increase of SRF from 40.0 μm (at 4 weeks) to 50.3 μm (at 7 weeks). Accordingly, CMT decreased from 281.0 μm (at 4 weeks) to 239.0 μm (at 20 weeks) in the first patient but increased from 202.0 μm (at 4 weeks) to 218.0 μm (at 7 weeks) in the second patient. On the other hand, subfoveal CT continued to decrease after the 4 weeks treatment duration in both of these patients. Subfoveal CT decreased from 370.7 μm (at 4 weeks) to 312.2 μm (at 20 weeks) in the first patient and from 370.0 μm (at 4 weeks) to 352.5 μm (at 7 weeks) in the second patient. BCVA was unchanged compared to pre-treatment in both of these patients.

### Adverse events

One patient withdrew from the study due to palpitations and headache following the first dose of the drug. Another patient withdrew after 2 weeks of treatment due to shortness of breath. Both instances were not believed by the study team and IRB to be caused by the drug. Among patients who completed the study, one patient developed hypertension after first week of using eplerenone. Leg cramps, mild headache, nausea, body ache, and ocular migraine occurred in 2 patients. All adverse effects resolved without discontinuing treatment. No significant changes in serum creatinine and potassium levels were observed (Table 3).

## Discussion

The primary outcome measure of this study was change in SRF height on OCT scans after 4 weeks of oral eplerenone treatment. SRF showed a tendency towards decrease (16.77%) after 4 weeks of treatment (94.18 ± 17.53 μm) as compared to baseline (113.15 ± 18.69 μm), although it was not statistically significant (p = 0.08). Similarly, Bousquet et al. did not find a significant change in SRF after 1-month of eplerenone treatment [16]. However, some studies demonstrated a significant decrease of SRF after 1 month of treatment with eplerenone [9, 12, 17]. In a double-blind, placebo-controlled study, Rahimy et al. demonstrated 62.81% decrease in SRF after 9 weeks of treatment with eplerenone as compared to baseline [13]. On the other hand, although Schwartz et al. found significant reduction in SRF as early as 1-week from start of eplerenone treatment as compared to baseline, they were not able to find a significant change in comparison with placebo [12].

In this study, CMT and subfoveal CT decreased significantly after 4 weeks of treatment as compared to baseline (7.05% and 6.6%, respectively). Previous studies have also found a clinically significant decrease of CMT under oral eplerenone treatment [12, 13, 16, 18]. Bousquet et al. reported significant decrease (30.11%) of CMT at 1-month follow up in comparison with baseline [16]. Suzuki et al., also reported significant dose-dependent decrease of CMT more at 3 months as compared to 1 month treatment [19]. Interestingly, Schwartz et al. did not find the CMT changes superior in the eplerenone group in comparison with placebo [12]. With regard to subfoveal CT, a significant decrease after 4 weeks of treatment has also been noted in previous studies [9]. However, Ghadiali et al. results are in contrast with our study, in which they showed decreased SRF, but not CMT and CT, in long term (12 months) follow up of CSCR patients who were treated with mineralocorticoid antagonists [20]. In addition, Swartz et al. were unable to find any significant change in CT during the follow up period despite observing decreased SRF and CMT in patients treated with eplerenone [12].

Visual acuity did not change significantly after 1 month of eplerenone treatment. It is notable that 7/13 (53.8%) of our patients had 20/20 vision before starting the treatment and among the 6 with worse than 20/20 vision, 4/6 (66.6%) had improvement. There was no significant correlation between SRF height or CMT and visual acuity. Following fluid resorption, visual changes may still be reported by patients for several months after the initial episode, suggesting that visual acuity may not be the best marker for visual impairment in these patients [21]. Intact RPE is significantly related to the improvement of visual acuity in CSCR patients treated with eplerenone [18]. Similar to our results, Schwartz et al. and Bousquet et al. did not find a clinically significant improvement in BCVA after 30 days of spironolactone treatment in patients with chronic CSCR [12, 22]. They attributed this to their low sample size and a high baseline BCVA [22], which might be true in our study as well. On the other hand, the most recent meta-analysis of randomized clinical trials with mineralocorticoid receptor antagonists shows a significant improvement in BCVA after 1 and 2 months of therapy as compared to placebo (− 0.05 logMAR; 95% CI − 0.07 and − 0.10 logMAR; 95% CI − 0.14 to − 0.06, respectively) [14]. Rahimy et al. also showed a significant improvement of visual acuity in 64% of patients treated with eplerenone for 9 weeks, from 20/50 to 20/43 (p = 0.04) [13]. Daruich et al. noted a significant change in BCVA after 6 months of treatment [9]. In addition, Zola et al. reported progressive improvement of visual acuity over 24 months follow up period [23]. These all suggest that a longer treatment period may result in better BCVA outcomes.

Non-responders, defined as patients who did not experience any change or who continued to accumulate more SRF throughout the treatment course, constituted 30.76% (4/13) of our study population. Four patients (30.7%) demonstrated an increase in SRF at the end of follow up as compared to the baseline. One of these patients remained on eplerenone for a total of 7 weeks and demonstrated a steady increase in SRF throughout the entire course. Our results are in line with previously reported data, which showed 30.8% [12] and 33.3% [24] of chronic CSCR patients were non-responders with eplerenone treatment. Longer disease duration was found to be the only contributing factor for non-responders [12]. Interestingly, Rajesh et al. found baseline BCVA as the strongest predicting factor for effectiveness of eplerenone [25]. The fact that those patients who were resistant to other previous therapeutic modalities such as focal laser therapy, intravitreal bevacizumab or PDT did not also responded to eplerenone treatment raises the importance of selecting suitable CSCR candidates for eplerenone therapy.

No study to date has reported life-threatening side effects of eplerenone. Fatigue [16], mild bowel irritation and myotonia [21] as well as diarrhea and dizziness [13] were reported before by CSCR patients, treated with eplerenone. In this study, hypertension, leg cramps, mild headache, nausea, body ache, and ocular migraine were observed in 3 patients during the study, which resolved without discontinuation of the drug.

This study has several limitations including the small sample size, not all subjects were treatment naïve, treatment duration was short (4 weeks), and there was no control group for comparison.

In conclusion, eplerenone treatment for a short period may facilitate a decrease in choroidal thickness and CMT in patients with CSCR. However, it does not decrease SRF height or improve visual acuity in a statistically significant manner. More prospective, randomized control studies with larger number of patients in both treatment and placebo arms are needed to better understand the role of eplerenone on the course and outcome of CSCR.

## Data Availability

The datasets used and/or analyzed during the current study are available from the corresponding author on reasonable request.

## Abbreviations

CSCR: chronic central serous chorioretinopathy
BCVA: best corrected visual acuity
OCT: optical coherence tomography
SRF: sub retinal fluid
CT: choroidal thickness
CMT: central macular thickness
RPE: retinal pigmented epithelium
ETDRS: Early Treatment Diabetic Retinopathy Study
logMAR: logarithm of the minimum angle of resolution
EDI: enhanced depth imaging
IRB: Institutional Review Board
